# Supplementary material for: Oligodendrocyte dynamics dictate cognitive performance outcomes of working memory training in mice
Source: Nat Commun. 2023 Oct 14;14:6499. doi: 10.1038/s41467-023-42293-4 (PMC10576739; doi:10.1038/s41467-023-42293-4)
Supplement: Supplementary file 1 — Supplementary Information [file 41467_2023_42293_MOESM1_ESM.pdf]

Supplementary Information

Oligodendrocyte dynamics dictate cognitive performance outcomes of working memory training in mice

Takahiro Shimizu[1] *, Stuart G Nayar[1] *, Matthew Swire[1], Yi Jiang[1], Matthew Grist[1], Malte Kaller[2], Cassandra Sampaio Baptista[2,$], David M Bannerman[3], Heidi Johansen-Berg[2], Katsutoshi Ogasawara[4], Koujiro Tohyama[5], Huiliang Li[1] and William D Richardson[1]

[1] Wolfson Institute for Biomedical Research, University College London, Gower Street, London WC1E 6BT, UK; [2] Wellcome Centre for Integrative Neuroimaging, Department of Clinical Neurosciences, John Radcliffe Hospital, University of Oxford, Oxford OX3 9DU, UK; [3] Department of Experimental Psychology, University of Oxford, Oxford OX1 3TA, UK; [4] Technical Support Center for Life Science Research and [5] Department of Physiology, Iwate Medical University, 1-1-1 Idaidori, Yahabacho, Shiwa-gun, Iwate 028-3694, Japan

[$] present address: Institute of Neuroscience and Psychology, University of Glasgow, 62 Hillhead Street, G12 8QB, UK

* joint first authors

Inventory

1. **Supplementary Figure S1**. *Behaviour of Myrf-cKO mice and comparison of male and female wild types.*

2. **Supplementary Figure S2.** *Proliferation and differentiation of OLPs in good- versus poor-performers.*

3. **Supplementary Figure S3.** *Working memory score correlates with training-induced OLP proliferation and differentiation.*

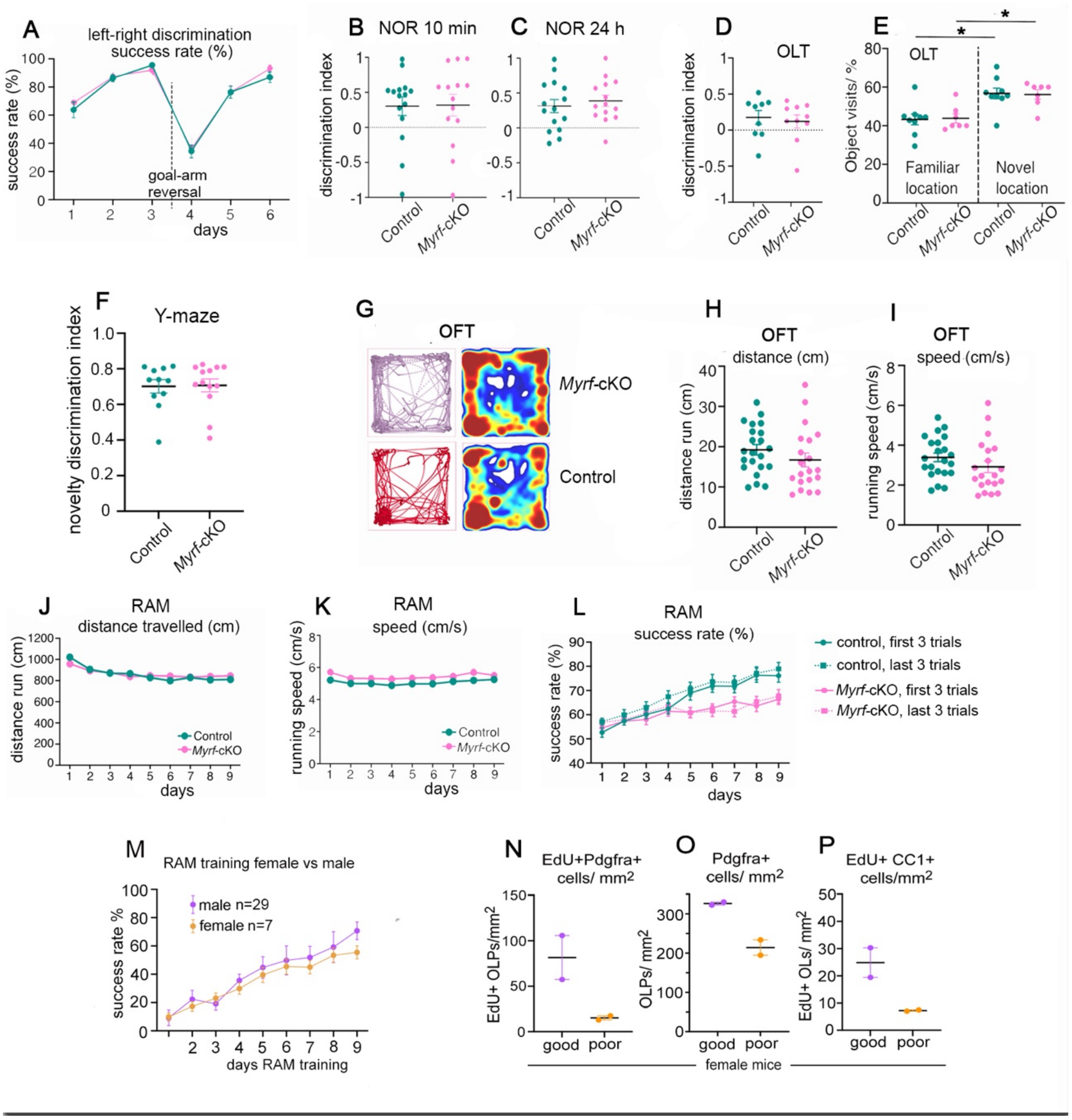

Supplementary Figure S1. **Behaviour of *Myrf*-cKO mice and comparison of male and female wild types.** (**A**) *Myrf*-cKO (n=12) and control mice (n=16) performed indistinguishably in a T-maze left-right discrimination task, both before and after reversal of the goal-arm, demonstrating normal reference memory formation and reversal learning in the absence of new OL generation (repeated measures 2-way ANOVA time x genotype p=0.85, F (5, 130) = 0.39; time p<0.0001, F (5, 130) = 52.4; genotype p=0.38, F (1, 26) = 0.78; Šídák's multiple comparisons test did not detect any significant differences). (**B**, **C**) There was no difference between *Myrf*-cKO (n=14) and controls (n=15) in the novel object recognition (NOR) discrimination index after either 10 min delay (*Myrf*-cKO, 0.32 ± 0.15; control, 0.30 ± 0.13) (**B**) or 24 h delay (*Myrf*-cKO, 0.39 ± 0.082; control, 0.31 ± 0.094) (**C**) (Kolmogorov-Smirnov non-parametric test, p=0.3 at 10 min, D=0.37, p=0.7 at 24 h, D=0.26). (**D**, **E**) There was no difference between groups in the novel object location (NOL) task

measured either by discrimination index (**D**) (*Myrf*-cKO, 0.12 ± 0.090, n=10; control, 0.18 ± 0.095, n=9, p=0.68, t=0.4, df=17) (unpaired Student's 2-tailed t-test) or frequency of visits to familiar or novel object locations (**E**) (familiar location: *Myrf*-cKO 43.8 ± 2.4%, n=7; control, 43.3 ± 2.8%, n=9. novel location: *Myrf*-cKO, 56.2 ± 2.4%, n=7; control, 56.8 ± 2.8%, n=9) (one-way ANOVA, F=8, df=28). Both *Myrf*-cKO and control groups visited the novel object location more frequently than the familiar object location [p=0.034 for control (familiar) vs control (novel); p=0.019 for *Myrf*-cKO (familiar) vs *Myrf*-cKO (novel)] (one-way ANOVA). (**F**) In the Y-maze test for spatial novelty preference and short-term spatial recognition memory, *Myrf*-cKO mice and controls spent similar times in the unfamiliar ("novel") arm versus the familiar ("other") arm, expressed as "novelty discrimination index" [time in novel arm / time in (novel+other) arms] (control: 0.70 ± 0.038, n=11. *Myrf*-cKO: 0.71 ± 0.036, n=13, p=0.99. Kolmogorov-Smirnov (KS) non-parametric test, D=0.18). (**G**) In the open field test (OFT), no obvious difference in the behaviour of *Myrf*-cKO (n=20) versus control (n=22) groups was evident in bird's nest maps (left) or heat maps (right). (**H**,**I**) In the OFT there were also no differences between groups either in distance travelled (**H**) (*Myrf*-cKO, 16.7 ± 1.7 cm; control, 19.3 ± 1.3 cm, p=0.24, t=1.2, df=40), or running speed (**I**) (*Myrf*-cKO, 2.9 ± 0.29 cm/s; control, 3.4 ± 0.22 cm/s, p=0.20, t=1.3, df=40) (unpaired Student's 2-tailed t-tests). (**J**,**K**) In the radial arm maze (RAM), neither the total distance travelled (**J**) nor the average running speeds (**K**) of *Myrf*-cKO and control mice were significantly different over the 9 days of testing (running speed: repeated measures 2-way ANOVA, time x genotype F(8, 376) = 3.7, time F(5.5, 256) = 35, genotype F(1, 47) = 0.089, Šídák's multiple comparisons test).. (**L**) Success rates in the RAM task separated into the first 3 and last 3 of the 6 trials of each day showed no inter-trial differences between *Myrf*-cKO (n=28) and control (n=29) groups (repeated measures 2-way ANOVA, time x genotype F(24, 880) = 3.5, time F(5.8, 635) = 50, genotype F(3, 110) = 5.1, Tukey's multiple comparisons test), indicating that the reason *Myrf*-cKO mice performed less well than controls was not because of increased interference – i.e. they did not confuse arm visits made in later trials with those made in earlier trials. (**M**-**P**) Performance and OL dynamics of female mice. (**M**) Female mice (phenotypically wild type, n=7) improved their performance over 9 days of RAM training, similar to male mice (n=29, same dataset as in Fig. 1C, D). Among the 7 females tested were 2 good-performers and 2 poor-performers; numbers of $EdU^{+}Pdgfra^{+}$ OLPs (**N**), total $Pdgfra^{+}$ OLPs (**O**) and $EdU^{+}CC1^{+}$ newly-formed OLs (**P**) were all elevated in the good-performers relative to the poor-performers, similar to male mice. Data in **A**-**M** are presented as mean ± s.e.m. (Student's two-tailed t-test) *p ≤ 0.05. Low "n" in **N**-**P** preclude statistical analysis. Source data are provided as a Source Data file.

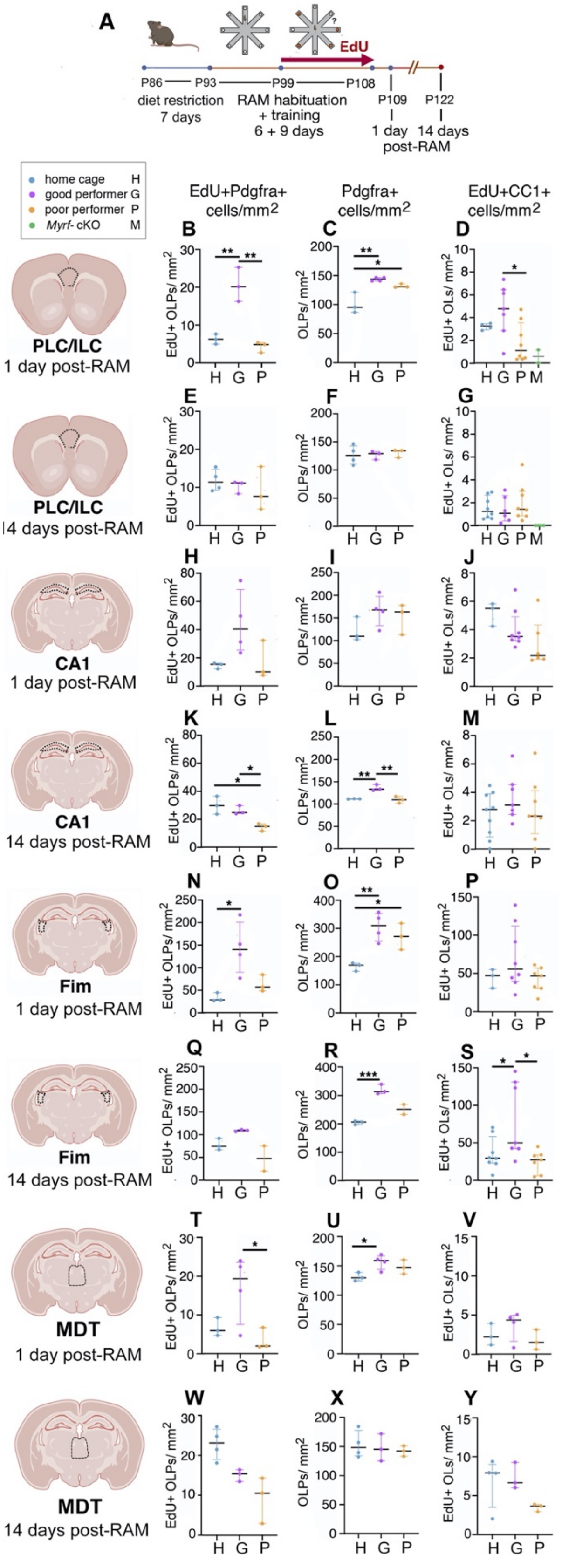

Supplementary Figure S2. Proliferation and differentiation of OLPs in good- versus poor-performers. (**A**) Experimental protocol. Mice were from our *Pdgfra-CreER*^{T2}*:Myrf*$^{(flox)}$

breeding colony. *Myrf*$^{(flox/flox)}$ and some *Myrf*$^{(flox/+)}$ mice received tamoxifen on days P60-63, as in Fig. 1A, some *Myrf*$^{(flox/+)}$ mice did not. Mice received EdU in their drinking water during radial arm maze (RAM) training and were perfusion-fixed 1- or 14-days post-training. RAM-trained mice were characterized as good- or poor-performers based on whether they achieved ≥10 or ≤5 "perfect trials", respectively, over the 9 days of RAM training. Home cage controls did not experience dietary restriction and were not exposed to the RAM at any time. (**B**-**D**) In the prelimbic/ infralimbic cortex (PLC/ ILC at 1-day post-RAM, the number-densities of proliferating OLPs (EdU$^+$ Pdgfra$^+$), and newly-formed OLs (EdU$^+$CC1$^+$) were increased in good-performers relative to poor-performers, similar to anterior cingulate cortex (ACC, main text and Fig. 3). (**E**-**G**) By 14-days post-RAM, number densities of OL lineage cells had returned to pre-training (home cage control) levels, also like ACC. (**H**-**M**) In hippocampal CA1 there were no significant changes in the densities of OL lineage cells at 1-day (**H**-**J**) or 14-days post-RAM (**K**-**M**), although there was perhaps a trend towards increased density of EdU$^+$ Pdgfra$^+$ recently divided OLPs in good-performers versus controls (**H**). (**N**-**S**) In the Fimbria (Fim), OL dynamics were similar to the anterior corpus callosum (CC, main text and Fig. 3), but less pronounced. At 1-day post-RAM there was a shift towards a higher density of EdU$^+$ Pdgfra$^+$ recently-divided OLPs (**N**) and a parallel upwards shift in the density of EdU$^+$ CC1$^+$ newly-differentiated OLs (**P**) in good-performers versus both poor-performers or home-cage controls; however, only the increases over home cage controls reached statistical significance. By 14-days post-RAM the density of EdU$^+$ Pdgfra$^+$ OLPs had returned close to control levels while the increased density of EdU$^+$CC1$^+$ OLs in good-performers versus controls persisted. (**B**-**Y**) x-axis labels are: H=home cage control, G=good performer, P=poor performer, M=*Myrf*-cKO, as also indicated in the key beneath panel (**A**). Data are presented as median ± 25%-75% interquartile range. p-values were determined by the Kruskal-Wallis non-parametric test, corrected for multiple comparisons using the Benjamini-Krieger-Yekutieli (BKY) false discovery rate test [89]. *$p \leq 0.05$, **$p \leq 0.01$, ***$p \leq 0.001$. See Supplementary data (Table S2) for full statistics. Source data are provided as a Source Data file. Drawings were created using BioRender.

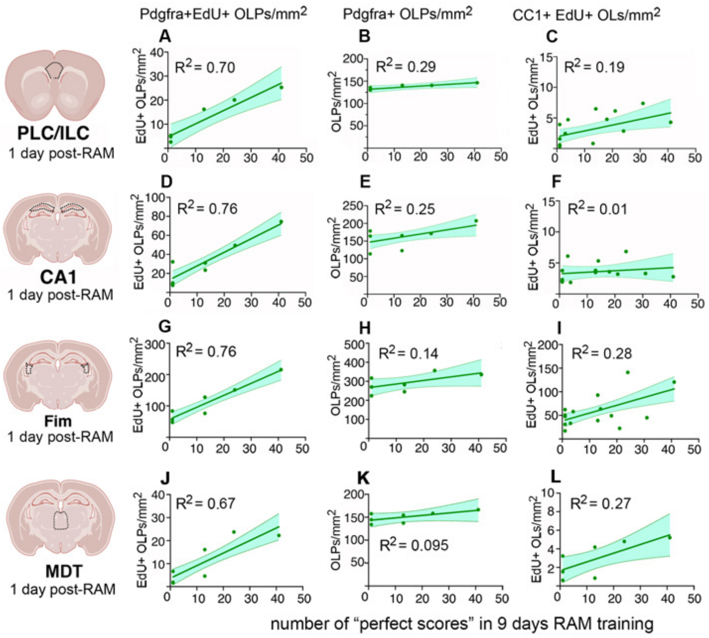

Supplementary Figure S3. Working memory score correlates with training-induced OLP proliferation and differentiation. At one day post-training, the working memory performance of individual mice in the radial arm maze (estimated by number of "perfect scores" during the 9 days of RAM training) correlates closely ($R^2 > 0.7$) with the number-density of proliferating OLPs (Pdgfra$^+$ EdU$^+$) in the prelimbic/infralimbic cortex (PLC/ILC, **A**) hippocampal CA1 (**D**) and fimbria (Fim, **G**) — but less so with the densities of newly-generated OLs (CC1$^+$ EdU$^+$) (**C**, **F**, **I**). Lines of best fit (simple linear, least-squares regression) are drawn with 95% confidence intervals; R^2 and n values are shown on graphs and in Supplementary data (Table S3), together with slopes and intercepts. Source data are provided as a Source Data file. Drawings were created using BioRender.
