# Supplementary material for: Oligodendrocyte dynamics dictate cognitive performance outcomes of working memory training in mice
Source: Nat Commun. 2023 Oct 14;14:6499. doi: 10.1038/s41467-023-42293-4 (PMC10576739; doi:10.1038/s41467-023-42293-4)
Supplement: Supplementary file 3 — Description of Additional Supplementary Files [file 41467_2023_42293_MOESM3_ESM.pdf]

Description of Additional Supplementary Files

Supplementary Data 1: Number-densities of OL lineage cells (cells/mm^2) following RAM training, comparing good-performers to poor-performers and home cage controls (median with interquartile range 25%-75%). Tabulated p-values refer to comparison with good-performers (Kruskal-Wallis non-parametric test with Benjamini-Krieger-Yekutieli correction for multiple comparisons). Uncorrected p-values are denoted "p" and corrected p-values as "q". p-values > 0.05 are regarded as non-significant (n.s.). This Table relates to Fig. 3.

Supplementary Data 2: Number-densities of OL lineage cells (cells/mm^2) following RAM training, comparing good-performers to poor-performers and home cage controls (median with interquartile range 25%-75%). Tabulated p-values refer to comparison with good-performers (Kruskal-Wallis non-parametric test with Benjamini-Krieger-Yekutieli correction for multiple comparisons). Uncorrected p-values are denoted "p" and corrected p-values as "q". p-values > 0.05 are generally regarded as non-significant (n.s.). Dashed lines indicate that low "n" value precludes statistical analysis. This table relates to Supplementary Fig. S2.

Supplementary Data 3: Lines of best fit (simple linear least-squares regression, Y=mX+c, where m is the slope and c the Y-intercept) and associated R2 and p-values, when number-densities of OL lineage cells (cells/mm2) are plotted against numbers of perfect trials attained by individual mice during RAM working memory training. (Relates to Fig. 4 and Supplementary Fig. S3.)

Supplementary Data 4: Lines of best fit (simple linear least-squares regression, Y=mX+c, where m is the slope and c the Y-intercept) and associated R2 and p-values, when number-densities of c-Fos+ ACC neurons (Fig. 7F) or average c-Fos fluorescence intensities (arbitrary units) (Fig. 7G) are plotted vs numbers of perfect trials attained by individual mice during RAM working memory training; or when densities of OL lineage cells in either the ACC or CC are plotted against numbers of perfect trials (Fig. 7H-K); or when densities of c-Fos+ ACC neurons are plotted vs densities of OL lineage cells in either the ACC or CC (Fig. 7L-O).

Supplementary Video 1: Control mouse on days 1 and 8 of the T-maze rewarded alternation task.

Supplementary Video 2: Good-performing mouse on days 1 and 9 of the RAM task.

Supplementary Video 3: Poor-performing mouse on days 1 and 9 of the RAM task.

Supplementary Video 4 : *Myrf*-cKO mouse on days 1 and 9 of the RAM task.
