# Supplementary material for: Oligodendrocyte dynamics dictate cognitive performance outcomes of working memory training in mice
Source: Nat Commun. 2023 Oct 14;14:6499. doi: 10.1038/s41467-023-42293-4 (PMC10576739; doi:10.1038/s41467-023-42293-4)
Supplement: Supplementary file 12 — Reporting Summary [file 41467_2023_42293_MOESM12_ESM.pdf]

nature portfolio

Corresponding author(s): William D. Richardson

Last updated by author(s): Aug 30, 2023

Reporting Summary

Nature Portfolio wishes to improve the reproducibility of the work that we publish. This form provides structure for consistency and transparency in reporting. For further information on Nature Portfolio policies, see our Editorial Policies and the Editorial Policy Checklist.

Statistics

For all statistical analyses, confirm that the following items are present in the figure legend, table legend, main text, or Methods section.

n/a	Confirmed	
☐	☒	The exact sample size (*n*) for each experimental group/condition, given as a discrete number and unit of measurement
☐	☒	A statement on whether measurements were taken from distinct samples or whether the same sample was measured repeatedly
☐	☒	The statistical test(s) used AND whether they are one- or two-sided<br>*Only common tests should be described solely by name; describe more complex techniques in the Methods section.*
☐	☒	A description of all covariates tested
☐	☒	A description of any assumptions or corrections, such as tests of normality and adjustment for multiple comparisons
☐	☒	A full description of the statistical parameters including central tendency (e.g. means) or other basic estimates (e.g. regression coefficient) AND variation (e.g. standard deviation) or associated estimates of uncertainty (e.g. confidence intervals)
☐	☒	For null hypothesis testing, the test statistic (e.g. *F*, *t*, *r*) with confidence intervals, effect sizes, degrees of freedom and *P* value noted<br>*Give P values as exact values whenever suitable.*
☒	☐	For Bayesian analysis, information on the choice of priors and Markov chain Monte Carlo settings
☒	☐	For hierarchical and complex designs, identification of the appropriate level for tests and full reporting of outcomes
☐	☒	Estimates of effect sizes (e.g. Cohen's *d*, Pearson's *r*), indicating how they were calculated

Our web collection on statistics for biologists contains articles on many of the points above.

Software and code

Policy information about availability of computer code

Data collection	no data collection software used
Data analysis	Statistics: Graphpad Prism 9.0; OriginPro 2023b(10.05) OriginLab; Immunofluorescence image analysis: NIH ImageJ 1.52r; MatLab R2022b Update 3 (9.13.0.2145394); Zen Blue 3.2; Zen Black 2.3 v14; Adobe Photoshop Elements 2023; Mouse tracking: Ethovision XT13.0.1220 Noldus; Acutal-Track 3.2.0 engine Actual Analytics; Video editing: Blender 3.3.1 LTS.

For manuscripts utilizing custom algorithms or software that are central to the research but not yet described in published literature, software must be made available to editors and reviewers. We strongly encourage code deposition in a community repository (e.g. GitHub). See the Nature Portfolio guidelines for submitting code & software for further information.

Data

Policy information about availability of data

All manuscripts must include a data availability statement. This statement should provide the following information, where applicable:

- Accession codes, unique identifiers, or web links for publicly available datasets
- A description of any restrictions on data availability
- For clinical datasets or third party data, please ensure that the statement adheres to our policy

All data (cell counts, node measurements, behavioural experiment results in Excel spreadsheets) will be made available freely on request.

Human research participants

Policy information about studies involving human research participants and Sex and Gender in Research.

Reporting on sex and gender	n/a
Population characteristics	n/a
Recruitment	n/a
Ethics oversight	n/a

Note that full information on the approval of the study protocol must also be provided in the manuscript.

Field-specific reporting

Please select the one below that is the best fit for your research. If you are not sure, read the appropriate sections before making your selection.

☒ Life sciences ☐ Behavioural & social sciences ☐ Ecological, evolutionary & environmental sciences

For a reference copy of the document with all sections, see nature.com/documents/nr-reporting-summary-flat.pdf

Life sciences study design

All studies must disclose on these points even when the disclosure is negative.

Sample size	We did not perform a priori power analyses to calculate the sample size for the experiments, because the size of the effects expected was unknown before doing the experiments. We did, however, perform sample size calculation post hoc for experiments where group differences were close to significant, using the web calculator (http://powerandsamplesize.com/Calculators/Compare-2-Means/2-Sample-Equality) with the observed mean ± s.d. of the control group, the apparent effect size, standard deviation of +/-12%, a power of 90% and p-value<0.05. For the behavioural time courses of Fig.1, a statistically significant effect of the observed magnitude on the final 3 days of the tests should be detectable with n=27 mice For the cell counts of Figs 3, S2, 5 we should be able to detect a significant difference, where it exists, with n=3 to n=6 mice per experimental condition, 3-5 sections per mouse.
Data exclusions	no data were excluded from analysis
Replication	Behavioural experiment data in Figs 1 and S1A-L are derived across 3-6 independent cohorts of mice. Fig S1M-P female data was conducted as one cohort and compared to the male data From Fig 1.<br><br>The cell count data in Figs 2, 3, 4, 5, 6, 7 and S2 are in most cases derived from two or three independent cohorts of mice; each cohort by itself as well as their combination gave a similar result (i.e. good performers made more OL lineage cells than poor performers or home cage controls). In addition, cell counts were self-consistent across brain regions (e.g. in ACC vs PLC/ILC, or in CC vs Fimbria).<br><br>The myelinated axon, node and paranode counts made in the EM are based on the sum of two independent cohorts that were processed, sent to Japan and analyzed on 2 separate occasions, as they became available. The separate cohorts both demonstrated a trend towards higher number-density of nodes/paranodes and higher number-density of myelinated axon cross-sections.<br><br>All experimental data replications are shown and were successful.
Randomization	In Figs 1 and S1, the breeding regimen was designed to generate roughly equal numbers of Myrf-cKO and control mice in the same litters. Therefore, all littermates were tested in the T-maze or RAM and only genotyped after the experiment. Figs 2,3,S2, 4, S3, 5, 6, 7: phenotypically wild-type mice were randomly assigned to home-cage control group or RAM-trained group, approximately one-third of the available mice in the home-cage control group. The RAM-trained group was separated post-hoc into good-and poor-performers based on their individual maze scores.
Blinding	Figs. 1, S1: Genotyping was performed after behavioural testing in all cases except for T-maze left-right discrimination, where genotype was determined before testing in order to assign roughly equal numbers of each genotype to left or right goal-arms. Figs. 2, 3, S2, 4, S3, 6: tissue sections were cut, mounted on glass slides and confocal images generated by SN; the image files were then transferred to MG who shuffled and re-named the images in an uninformative manner so that all three groups were intermingled. MG then passed the images back to SN for cell-counting, after which MG decoded the image labels and SN plotted the data. Fig 5 C-D and Fig 7 tissue sections were cut by SN and passed on to MS, blind to all tissue, who stained, generated confocal images and counted cell numbers and/or intensity. MS provided SN with the data to decode and plot the graphs. Fig 5H-J: phenotypically wild- type mice were randomly assigned to home-cage control or RAM-trained groups and the latter group separated post-training into good- or poor-performers. Fixed brains from good-performing mice and an equal number of home cage controls were labeled uninformatively and sent to Japan for EM analysis, which was performed blind to whether the mice were from control or experimental groups. The data were then decoded in the UK.

Reporting for specific materials, systems and methods

We require information from authors about some types of materials, experimental systems and methods used in many studies. Here, indicate whether each material, system or method listed is relevant to your study. If you are not sure if a list item applies to your research, read the appropriate section before selecting a response.

Materials & experimental systems

n/a	Involved in the study
	☒ Antibodies
☒	☐ Eukaryotic cell lines
☒	☐ Palaeontology and archaeology
	☒ Animals and other organisms
☒	☐ Clinical data
☒	☐ Dual use research of concern

Methods

n/a	Involved in the study
☒	☐ ChIP-seq
☒	☐ Flow cytometry
☒	☐ MRI-based neuroimaging

Antibodies

Antibodies used	Primary antibodies were anti-Olig2 (rabbit, Merck AB9610, 1:500, Lot # 3857662), monoclonal APC clone CC-1 (mouse, Calbiochem OP80, 1:200, Lot # 3783180), anti-YFP (chicken, Aves labs, GFP-1020, 1:1,000, Lot # GFP3717982), anti-Pdgfra (rabbit, Cell Signalling Technology 3164S, 1:200, Lot # 6), anti-Caspr clone K65/35 (mouse, Merck MABN69, 1:300, Lot # 3823691), anti-NaV1.6 (rabbit, Alomone ASC-009,1:500, Lot # ASC009AC3102), anti-cFos (rabbit, Abcam ab190289, 1:1000, Lot # 1026805-1). Secondary antibodies were Alexa-variants (Invitrogen) anti-chicken 488 nm (goat, Invitrogen, A11039, 1:1,0000, Lot # 2566343), anti-rabbit 488 nm (donkey, Invitrogen, A21206, 1:1,000, Lot # 2541645), anti-rabbit 568 nm (donkey, Invitrogen, A10042, 1:1,000, Lot # 2207536), anti-rabbit 647 nm (donkey, Invitrogen, A31573, 1:1,000, Lot # 2577247), anti-mouse 568 nm (donkey, Invitrogen, A10037, 1:1,000; Lot # 2555709), anti-mouse 647 nm (donkey, Invitrogen, A31571, 1:500, Lot # 2420713). These are well-known and reliable reagents with which we also have a great deal of experience.
Validation	All antibodies used in this study are standard reagents in oligodendrocyte research and have been in everyday use and published by many members of our laboratory over many years. Specifically Olig2, CC1, YFP, Pdgfra have been published by us (McKenzie et al., 2014, Science. PMID: 25324381; Xiao et al., 2016, Nat. Neurosci. PMID: 27455109) and are commercially validated. The node/paranode antibody markers anti-Nav1.6 and anti-Caspr are commercially validated and our protocol followed a previous publication (Arrancibia-Carcamo et al., 2017, eLife, PMID: 28130923). The immediate early neuronal activity marker, anti-cFos is commercially validated and is an established and commonly-used reagent that gives a distinctive pattern of immunolabelling (Li et al., 2023, J. Neurosci. PMID: 37599352; Jovanovic et al., 2023, Nat. Comms. PMID: 37582805).

Animals and other research organisms

Policy information about studies involving animals; ARRIVE guidelines recommended for reporting animal research, and Sex and Gender in Research

Laboratory animals	All mice were aged 60-120 days postnatal, on a mixed C57b6/CBA/129 strain. Transgenic mice included: Pdgfra-CreERT2:Myrf-flox:Rosa26-YFP that were homozygous for Pdgfra-CreERT2 and Rosa26-YFP and either heterozygous or homozygous of Myrf-flox, and Pdgfra-CreERT2:TaumGFP that were heterozygous for both Pdgfra-CreERT2 and Tau-mGFP. Pdgfra-CreERT2 mice were generated in house and are available upon request. Myrf-flox mice were obtained from Ben Emery and are available from Jackson Laboratories (JAX: 010607). Tau-mGFP were obtained from Sylvia Arber and are commercially available from Jackson Laboratories (JAX: 021162). Rosa26-YFP mice are available from Jackson Laboratories (JAX: 006148).<br><br>Mice were predominantly group housed post-weaning. One week prior to radial maze training mice were individually housed to ensure appropriate diet restriction could be maintained throughout all experiments. Ambient temperature and humidity in the behavioural rooms and barn were maintained at around 21°C and 55%, respectively.
Wild animals	No wild animals were used in this study
Reporting on sex	Most of our experiments were conducted with male mice, to avoid potentially increased individual variation across the female reproductive cycle. However, we repeated some of our experiments using female mice and saw non-significant differences between sexes. Our previous studies (e.g. complex wheel-running experiments: McKenzie et al., 2014, Science 346, 318-322) has shown non-significant differences between male and female cohorts.
Field-collected samples	No field collected samples were used in this study
Ethics oversight	Mouse experiments were pre-approved by the UCL Animal Welfare and Ethical Research Board (AWERB) and by the Home Office of the UK Government, through the issue of a Project Licence under the UK Animals (Scientific Procedures) Act 1986 and subsequent amendments.

Note that full information on the approval of the study protocol must also be provided in the manuscript.
